# The Effect of Intradialytic Exercise on Cognition in Renal Patients Undergoing Hemodialysis: An Updated Systematic Review of Randomized Controlled Trials

**DOI:** 10.3390/healthcare13233016

**Published:** 2025-11-21

**Authors:** Andreas Mavrommatis, Nicos Mitsides, Myrtani Pieri, Eleni P. Andreou, Giorgos K. Sakkas, Kyproula Dimitriou, Michalis Spartalis, Maria Arsali, Themis Christofi, Theophanis Theophanous, Bettina Wollesen, Georgios M. Hadjigeorgiou, Christoforos D. Giannaki

**Affiliations:** 1 Department of Life Sciences, School of Life and Health Sciences, University of Nicosia, 2417 Nicosia, Cyprus; mavrommatisandreas@hotmail.com (A.M.); pieri.m@unic.ac.cy (M.P.); andreou.el@unic.ac.cy (E.P.A.); gsakkas@uth.gr (G.K.S.); 2 Research Centre for Exercise and Nutrition (RECEN), University of Nicosia, 2417 Nicosia, Cyprus; 3 Medical School, University of Cyprus, 1678 Nicosia, Cyprus; mitsides.nicos@ucy.ac.cy (N.M.); hadjigeorgiou.georgios@ucy.ac.cy (G.M.H.); 4 School of Physical Education, Sport Science and Dietetics, University of Thessaly, 42100 Trikala, Greece; 5 Hemodialysis Unit, General Hospital of Nicosia, 2031 Nicosia, Cyprus; k.dimitriou@shso.org.cy (K.D.); m.spartalis@shso.org.cy (M.S.); m.arsali@shso.org.cy (M.A.); them.christofi@shso.org.cy (T.C.); t.theophanous@shso.org.cy (T.T.); 6 Institute of Movement Therapy and Movement-Oriented Prevention and Rehabilitation, German Sports University Cologne, 50933 Cologne, Germany; b.wollesen@dshs-koeln.de

**Keywords:** aerobic exercise, resistance exercise, cognitive function

## Abstract

**Background/Objectives**: Hemodialysis patients are disproportionately affected by impaired cognitive function in comparison to the general population. This systematic review aims to update and expand the current evidence regarding the effects of IET interventions on global cognition and specific cognitive domains, such as executive function, processing speed, and attention. **Methods**: A comprehensive search was conducted across three databases (PubMed, Scopus, and EBSCO) from database inception to 24 August 2025 for randomized controlled trials examining the effects of intradialytic exercise training on cognitive function, using combinations of the following search terms: hemodialysis, dialysis, dialy*, physical exercise, physical activity, exercise, activity, activit*, cognition, cognitive, and cognit*. Included studies were assessed for risk of bias and methodological quality using the Jadad Scale and NHLBI tools. **Results**: Seven studies were included in this review, encompassing (n = 332; 60.4% male) hemodialysis patients aged from 48 to 74.9 years. In comparison to standard care, IET significantly improved global cognition and specific cognitive domains. Regarding global cognitive function, interventions regardless of exercise type, which were performed thrice weekly over 12 to 16 weeks, significantly improved scores in (n = 4; 57%) studies using the Montreal Cognitive Assessment (MoCA) and in (n = 1; 14%) study using the Mini Mental State Examination. Regarding specific cognitive domains, aerobic exercise performed thrice weekly for 12 weeks were associated with statistically significant improvements in the following: executive function scores (n = 2; 29%), studied using the Trail Making Test (TMT) Part-B and TMTB-A; psychomotor processing speed (n = 1; 14%), studied using TMT-A and Symbol Digit Modality Test (SDMT); and alertness (n = 1; 14%), studied using the Test of Attentional Performance (TAP) test. **Conclusions**: The collective evidence confers that IET is an effective intervention that may moderately improve global and domain-specific cognitive function or, at the very least, serve in a protective capacity to stem potential future cognitive decline in this population. However, further large-scale randomized controlled trials that place emphasis on standardized reporting of exercise intervention characteristics and cognitive outcome measures are necessary to inform clinical practice.

## 1. Introduction

Chronic kidney disease (CKD) is defined by “abnormalities of kidney structure or function, present for a minimum of 3 months” [1]. In lieu of kidney transplantation, hemodialysis (HD) constitutes the most widely utilized modality of kidney replacement therapy (KRT) among end-stage kidney disease (ESKD) patients [2]. Across the European Union, the prevalence of individuals receiving HD is estimated to reach approximately 50 million [3], with an annual incidence of newly diagnosed patients requiring HD ranging from 81 to 195 cases per million population [4]. Advancements in renal medicine, alongside the effective management of comorbidities, have markedly contributed to life expectancy and reduced patient burden in those who are newly diagnosed [5]. In spite of this, HD patients remain disproportionately burdened by systemic multimorbidity, generally resulting in progressive deterioration of physical, mental, and social capacity [6].

Among these complications, cognitive impairment has emerged as a particularly prevalent and debilitating consequence [7]. Depending on the assessment tools and dialysis treatment, the prevalence of general, undefined cognitive impairment in patients receiving HD is very high, ranging from 34% to 87% [8,9]. In comparison, the global prevalence of cognitive impairment in non-CKD adults lies between 3% and 7% [10]. The etiopathogenesis of impaired cognitive function in HD patients appears to be multifactorial, involving both conventional (i.e., vascular) and renal-disease-specific mechanisms, along with treatment-related complications [11]. Synoptically, cognitive impairment adversely affects a broad spectrum of cognitive domains, including memory, concentration, processing speed, executive functions, and visuospatial ability [12]. It is understood that even mild cognitive impairment in HD patients has the potential to significantly impair health-related quality of life (HRQOL), ominously tripling the risk of associated morbidity and mortality in comparison to HD patients without cognitive impairment [13].

Cognitive impairment in individuals with CKD is thought to arise from a combination of factors, including alterations in vascular function and exposure to uremic toxins [12]. This makes pharmacological efforts to improve cognitive function in this population even more challenging, prompting parallel investigations into potential non-pharmacological approaches [14]. Thus, in the last decade, there has been a growing interest in the effects of non-pharmacological interventions on cognitive performance and the incidence of dementia, principally concerning modifiable lifestyle risk factors such as increasing physical activity participation through intradialytic exercise [15].

Numerous systematic reviews and meta-analyses have indicated profound benefits associated with physical activity or exercise training in HD patients: (1) physiological improvements related to functional capacity, body composition, cardiorespiratory fitness, bone mineral density, inflammation, sleep, and HRQOL [16,17,18,19,20]; (2) improved psychological well-being and mental-health-related outcomes affecting quality of life, such as symptoms of anxiety, depression [21,22], and perceived fatigue [23], with intradialytic exercise further recognized to improve dialysis efficacy [24,25].

However, evidence addressing the effects of exercise on cognitive function in HD patients remains limited [26]. Exercise, as shown in non-CKD populations, can improve cognitive function [27]. Considering that in CKD patients, it has generally demonstrated very beneficial effects across various health-related parameters mentioned above, it is important to investigate whether it can also support cognitive function. Exercise can be performed in various forms (e.g., aerobic, strength, flexibility) or as a combination of these forms, with different intensities, frequencies of exercise sessions, and varying duration of each session [28,29]. Each type of exercise has its own characteristics, as do the adaptations that may occur following the patient’s regular participation in an exercise program [29,30]. In the general population, Yang and colleagues (2023) conclude that the most effective exercise interventions for improving cognitive performance are of a hybrid design (aerobic plus resistance component at the same session), short in duration, performed at moderate-to-high intensity workload, and attended frequently by participants [31]. However, we should note that the mechanisms by which exercise can improve cognitive function in HD patients remain relatively unexplored.

The aim of this systematic review is to investigate and evaluate the impact of intradialytic exercise on global cognition and various domains of cognitive function in HD patients. The main research questions sought are: (1) Which interventions can be identified as effective in improving cognitive performance in HD patients? (2) Which cognitive domains were addressed in these studies? (3) Which exercise training characteristics are required to gain positive effects on cognition? The findings of this review are intended to provide an updated synthesis of the available evidence.

## 2. Materials and Methods

### 2.1. Eligibility Criteria

Eligible randomized controlled trials (RCT’s) considered for inclusion in this systematic review were required to meet the following criteria: (1) publication in peer-reviewed journals; (2) written in the English language; (3) explicitly involving end-stage kidney disease patients ≥18 years of age receiving HD treatment; (4) protocols which specified an intradialytic exercise training intervention component; (5) use of validated tools to assess outcome measures for global cognition [e.g., Montreal Cognitive Assessment (MoCA), the Mini Mental State Exam (MMSE), or the Modified Mini Mental State Exam (3MS)] and/or domain specific cognitive ability [e.g., The Trail Making Test (TMT) Parts A and B, Symbol Digit Modality Test (SDMT), Test of Attentional Performance (TAP), Digit Symbol Substitution Test (DSST), or Rey Auditory/Verbal learning Test (RAVLT)].

Studies were excluded where (1) participants were diagnosed with end-stage kidney disease but did not receive HD treatment (i.e., peritoneal dialysis or recipients of kidney transplantation); (2) the exercise training intervention did not occur during the HD treatment session (i.e., inter-dialytic exercise training between routine hemodialysis treatments).

Based on these conditions, the PICOS (patient population, interventions or exposure, comparator group, outcome or endpoint, and study design) search tool was employed to generate keywords (Table 1) for the systematic search of electronic publication databases [32].

### 2.2. Information Sources

A comprehensive systematic search of several electronic academic publication databases was conducted, specifically retrieving articles from PubMed, EBSCO, and SCOPUS, published in English from database inception to 24 August 2025. To exhaust the discovery of any potential past and future studies, manually searching through the reference lists of the included studies and citation tracking were key in the identification of further studies potentially overlooked by the preceding electronic database searches. In particular, the reference lists of retrieved randomized controlled trials were then manually searched to identify additional past studies. Finally, the process of citation tracking was conducted through the Google Scholar website for key studies that were identified during the database searches. This involved examining articles that had cited these key studies, as well as checking for their inclusion in other systematic reviews. Language restrictions were applied during the citation tracking search process to only include studies published in the English language. The systematic literature search was carried out from [5 August 2025] to [24 August 2025] to encompass studies published within the defined time frame of interest.

### 2.3. Search Strategy

The search strategy employed a combination of keywords and synonyms generated from the PICOS tool (Table 1) to generate “Title and Abstract” and “Medical Subject Headings” (MeSH) terms where applicable using the MeSH Advanced Search Builder tool (https://www.ncbi.nlm.nih.gov/mesh/advanced (accessed on 13 November 2025)). The Boolean operators “OR” and “AND” were employed to comprehensively combine search terms, which defined the greatest possible number of definitions associated with HD (patients), cognitive function (outcomes), and exercise (intervention). The “asterix” symbol (*) attached to the stem of select words signifies a wildcard search of any words that may contain letters preceding the asterix. The comprehensive search algorithm for each database is presented in Table 2.

### 2.4. Study Selection and Data Extraction

The retrieved articles from each of the above-mentioned electronic database searches, past reference lists manual searching, and citation tracking actions were exported into the RefWorks (https://refworks.proquest.com/ (accessed on 13 November 2025)) citation manager software suite. An automated RefWorks search for duplicates using “exact match” criteria was also carried out for the collective library of articles retrieved during the wider search strategy.

An independent review was carried out by two researchers of the title and abstract of each eligible article meeting the inclusion criteria for this systematic review. Throughout this process, articles not meeting the explicit inclusion criteria were manually excluded. Then, studies were inspected with a full-text review, ensuring that the full inclusion criteria were met, especially methodological criteria specific to the intervention delivery type (i.e., intradialytic exercise training) and outcome measures specifically validated in the measurement of cognitive function. In summary, the following data were targeted for extraction: authors, year of publication, country in which the research was conducted, sample size in intervention and control groups, mean age of participants in intervention and control groups, participant sex, exercise training intervention (modality, frequency, intensity, duration and length of intervention period), control group intervention, measures of cognitive function, and results within and between group changes.

### 2.5. Study Quality and Risk of Bias

The likelihood of bias in the included studies was evaluated using the Jadad score (Table 3) [33]. The Jadad scale is a valid, widely used, and internationally recognized instrument for evaluating randomized controlled trials, particularly in intervention-based research regarding exercise rehabilitation [34]. The quality of studies screened for inclusion in this systematic review was determined using the National Heart, Lung, and Blood Institute’s (NHLBI) “Quality Assessment of Controlled Intervention Studies” tool (Table 4) (https://www.nhlbi.nih.gov/health-topics/study-quality-assessment-tools, accessed on 13 November 2025) which grades study quality across the following fourteen categorical questions: (1) Was the study described as randomized, a randomized trial, a randomized clinical trial, or an RCT? (2) Was the method of randomization adequate (i.e., use of randomly generated assignment)? (3) Was the treatment allocation concealed (so that assignments could not be predicted)? (4) Were study participants and providers blinded to treatment group assignment? (5) Were the people assessing the outcomes blinded to the participants’ group assignments? (6) Were the groups similar at baseline on important characteristics that could affect outcomes (e.g., demographics, risk factors, co-morbid conditions)? (7) Was the overall dropout rate from the study at endpoint 20% or lower of the number allocated to treatment? (8) Was the differential drop-out rate (between treatment groups) at endpoint 15 percentage points or lower? (9) Was there high adherence to the intervention protocols for each treatment group? (10) Were other interventions avoided or similar in the groups (e.g., similar background treatments)? (11) Were outcomes assessed using valid and reliable measures, implemented consistently across all study participants? (12) Did the authors report that the sample size was sufficiently large to be able to detect a difference in the main outcome between groups with at least 80% power? (13) Were outcomes reported or subgroups analyzed prespecified (i.e., identified before analyses were conducted)? (14) Were all randomized participants analyzed in the group to which they were originally assigned, i.e., did they use an intention-to-treat analysis? Risk of bias and study quality assessments were both independently evaluated by two reviewers; discrepancies in ratings between reviewers were resolved through discussion, and where necessary, a third reviewer was consulted for arbitration.

This particular review is not registered in an international systematic review registry.

## 3. Results

### 3.1. Literature Search and Characteristics of Included RCTs

**Figure 1:**
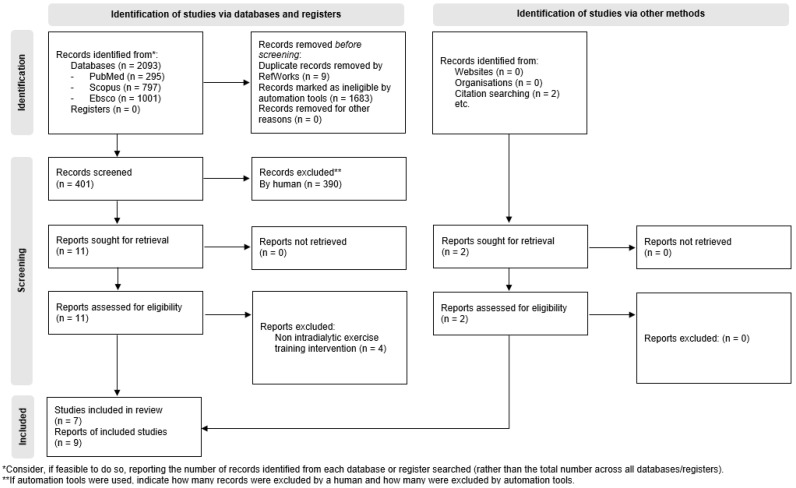
PRISMA flow diagram of study selection process.

The initial search strategy of electronic databases identified n = 2093 studies. Of these, n = 2026 were marked as ineligible and excluded by electronic database automation tools. Of the n = 6 records screened, just n = 5 studies met the eligibility criteria for inclusion in this systematic review. These studies were associated with further n = 562 records identified through manually tracking past and future citations from reference lists and Google Scholar, yielding another n = 2 studies eligible for inclusion. In total, n = 7 randomized controlled trials were included in this systematic review investigating the effects of intradialytic exercise on cognitive function. The process conformed to the PRISMA guidelines for systematic review reporting [44]; an illustrated summary of this process flow is presented in Figure 1 below.

### 3.2. Study Characteristics

All the included studies were conducted fairly recently, with over two-thirds, n = 5 (71%), published since 2020; the remaining n = 2 (29%) studies were published within this past decade. Cumulatively, (n = 332; mean age between 48 and 74.9 years; 60.4% male) HD patients were recruited across these seven studies, (n = 165) were randomized into intervention groups which received intradialytic exercise training (alone or in combination with a cognitive training component), and n = 129 formed the control groups which received standard care only. The remaining n = 38 participants received intradialytic cognitive exercise training only as part of the studies by McAdams, DeMarco and colleagues, and Ghildayal and colleagues. The intradialytic physical exercise modalities included aerobic (cycling) and strength training (resistance band), administered thrice weekly over twelve to twenty-four weeks (Table 5).

The intervention effects of intradialytic physical exercise training interventions on the outcomes of global cognitive function and domain-specific outcomes were evaluated across the included studies. Specifically, a total number of n = 7 studies examined the effects of intradialytic exercise training interventions on global cognitive function using the MoCA [35,36,37,39,41], the MMSE [43], or the 3MS [42,43]. The MoCA is a validated screening tool that detects mild cognitive impairment in clinical populations, including chronic kidney disease, especially across the cognitive domains associated with short-term memory, visuospatial abilities, executive functioning, attention and concentration, working memory, language, and orientation to time and place. The test is scored out of a maximum of 30 points; typically, a score of ≤25 is indicative of the presence of mild cognitive impairment [45]. The MMSE is another validated and widely used screening tool determining the degree of global cognitive impairment in chronic kidney disease, briefly assessing the performance in tasks involving the specific cognitive domains of immediate memory, attention, calculation, temporal and space orientation, language and visual constructional ability, and word recollection. The test is scored out of a maximum of 30 points, with normal cognitive function scores being > 24 points, mild cognitive impairment scores between 19 and 24 points, and severe cognitive impairment scores between 10 and 18 points [46]. The 3MS is referred to as a modified and expanded ‘update’ to the MMSE, which grades global cognition on a scale of (0–100). It has been reported to sample a broader spectrum of cognitive functions with greater sensitivity over the MMSE, thus informing metrics of greater validity and test–retest reliability [47].

Regarding changes within specific cognitive domains, a total number of n = 3 studies also examined the effects of intradialytic exercise training on specific cognitive outcomes using validated tools, such as executive function, psychomotor and processing speed, and attention and alertness [35,38,42]. The Symbol Digit Modality Test (SDMT) is a validated clinical assessment that measures performance of neurocognitive tasks associated with the cognitive domain of processing speed; graded on a scale of (0–110), derived from the number of correct substitutions participants make over a 90 s window [48]. The Trail Making Test Part-A (TMTA) and the Trail Making Test Part-B (TMTB) measure psychomotor speed and executive function, respectively [49]. The tests are scored in time, measured in seconds, where the fastest time indicates better cognitive function. Inferencing executive function ability is derived from the resulting time in seconds when the time to complete TMTA is subtracted from the time it took to complete TMTB (TMTB−TMTA) [42]. The Test of Attentional Performance (TAP) is a computerized assessment that evaluates sustained, selective, divided, and alertness-related components of attention in response to various tasks and stimuli [50]. The Rey Auditory/Verbal Learning Test (RAVLT) is a neuropsychological screening test that evaluates verbal learning and memory by measuring participants’ immediate and delayed ability to recall a list of spoken words [51]. The Digit Symbol Substitution Test (DSST) is a neuropsychological assessment that evaluates cognitive domains which are critical for daily functioning, namely processing speed, attention, working memory, visual–motor coordination, and cognitive flexibility [52]. The test is scored by the total number of correct symbol–digit matches during a time-limited window with possible scores ranging between 0 and 100; lower scores hint at impaired executive functions. A comprehensive summary of the included studies is presented in Table 5, whilst the specific exercise training characteristics employed in each of the seven intradialytic exercise protocols are summarized in Table 6.

Collectively, the majority of included studies in this review reveal a positive effect of intradialytic exercise interventions on cognitive performance. Ghildayal and colleagues (2025) [35] demonstrated that a 12-week intervention combining moderate to vigorous intensity aerobic exercise performed for 30 min thrice weekly, and cognitive training significantly improved global cognitive performance scores in the MoCA in comparison to standard care at 3 months. However, no significant differences were reported at 3 months in the cognitive domains of executive function, processing speed, or verbal learning and memory between groups. Feng and colleagues (2025) [36] demonstrated that a 12-week intervention of low-to-moderate intensity resistance exercise performed for 30–40 min thrice weekly produced significant improvement in global cognition. Specifically, within-group analysis revealed a significant improvement in MoCA scores for the exercise group between baseline and post-intervention (t = −9.257, *p* < 0.001) compared to standard care (t = −1.888, *p* = 0.069), with a significant group-by-time interaction (t = 5.047, *p* < 0.001). Likewise, Saputrana and colleagues (2024) [43] demonstrated that a 12-week intervention of low to moderate intensity aerobic exercise performed for 30 min twice weekly also produced significant improvement in global cognition. Within-group analysis revealed a significant improvement in MoCA scores for the exercise group between baseline and post-intervention (*p* < 0.001) compared to standard care (*p* = 0.468), with a significant group-by-time interaction (*p* < 0.001). Over a series of three reports, Bogataj and colleagues (2023/2024) [38,40] investigated the global and domain-specific effects of moderate intensity aerobic exercise performed for 30 min in combination with cognitive training, thrice weekly over 12 weeks. Study A examined the cognitive domain of attention using the TAP Test, revealing selective intervention effects. Specifically, within-group analysis identified a statistically significant decline in the standard care group’s alertness scores (*p* = 0.040), with no significant change reported within the intervention group between baseline and post-intervention. Whilst a statistically significant group-by-time interaction effect was also reported for alertness scores in favor of the intervention group. Conversely, no significant changes within or between groups were revealed for either selective or divided attention subsets, respectively. Study B investigated broader cognitive improvements through processing speed and global cognition, revealing significant group-by-time interaction for both SDMT (*p* < 0.001) and MoCA (*p* < 0.001) scores in favor of exercise, respectively. Study C examined executive function within group analysis of the exercise intervention group, which indicated a significant improvement across all TMT variant scores (TMT-A, TMT-B, TMTB-A), while the control group exhibited significant deterioration in executive function. Significant group-by-time interaction effects were also reported across all TMT variant scores of executive function performance in favor of the intervention group. The study by Nakamura-Taira and colleagues (2021) [41] investigated the effect of a 24-week intervention of low-intensity resistance exercise performed 25–30 min three times per week on global cognitive function. Within-group analysis revealed slight, yet non-significant changes in MoCA scores within both groups between baseline and post-intervention, with between-group comparisons also demonstrating no significant difference on global cognitive function (*p* > 0.05). Stringuetta Belik and colleagues (2018) [43] demonstrated that a 16-week intervention of moderate-to-high intensity aerobic exercise performed for 30–45 min thrice weekly revealed significant improvements in global cognition in the MMSE. Within-group analysis revealed significant improvement in MMSE scores for the exercise group between baseline and post-intervention (*p* < 0.001), with a significant group × time interaction in favor of the exercise group (*p* = 0.001). Finally, McAdams De-Marco and colleagues (2018) [42] investigated the effects of a 12-week aerobic exercise intervention performed for a modest 20 min three times per week on global cognitive function and executive function. Specifically, no significant differences were revealed within groups for global cognitive function with respect to 3MS scores between baseline and post-intervention. Similarly, no significant change was reported within groups for executive function, except for the presence of a significant decline between baseline and post-intervention concerning TMT-B scores within standard care. Between-group comparisons revealed a significant improvement favoring the exercise group over standard care only on TMT-B (*p* = 0.30), with no further statistically significant between-group differences observed for global cognitive status (3MS), processing speed (TMT-A), or (TMT B–A) (*p* > 0.05). In summary, the results of this particular systematic review reveal the positive effect of intradialytic exercise training on both global cognitive function scores and on specific domains such as executive function and alertness.

## 4. Discussion

Evidence from recent systematic reviews and meta-analyses reveals compelling evidence that both intradialytic and interdialytic exercise interventions performed for at least 30 min three times per week over 16 weeks significantly improve cognition in hemodialysis patients [53,54]. The outcomes of the current systematic review indicate a moderate beneficial effect on global cognitive scores and specific domains such as executive function and alertness, although variability in cognitive measures and exercise protocols contributes to some heterogeneity in reported outcomes. Specifically, the form that appears to be most effective for improving cognitive performance in HD patients is aerobic exercise. Regarding the different domains, the most consistently affected domains were global cognition, attention, and executive function, suggesting that exercise supports higher-order processing. Finally, concerning exercise training characteristics, it seems that cognitive improvement is most likely during moderate-to-high-intensity aerobic exercise performed for 30–45 min, three times per week, over at least 12 weeks.

Regarding global cognitive function, n = 5 (71%) studies found that in comparison to standard care intradialytic exercise training interventions, regardless of exercise modality but especially, those performed thrice weekly and lasting between 12 and 16 weeks were associated with a statistically significant improvement in MoCA [35,36,37,38] and n = 1 in MMSE [43] scores, respectively. Regarding specific cognitive domains, intradialytic aerobic exercise interventions performed thrice weekly for 12 weeks were associated with statistically significant improvements in the following: executive function scores in n = 2 (29%) studies using the TMT-B and TMTB-A [40,42]; psychomotor processing speed in n = 1 (14%) study using TMT-A and SDMT [38]; and alertness in n = 1 (14%) study using the TAP test [38], respectively.

In recent years, there has been considerable interest in the mechanisms through which exercise may help improve cognitive function. The cognitive benefits of exercise in HD patients are likely multifactorial, involving both direct effects on brain physiology and indirect effects through improved mental health, sleep, vascular function, and metabolic control. However, it should be noted that, currently, there is limited published data specifically addressing these mechanisms in HD patients. For example, exercise enhances cerebral blood flow and oxygen delivery, counteracting dialysis-related cerebral hypoperfusion [43]. It also stimulates the release of neurotrophic factors, such as brain-derived neurotrophic factor (BDNF), which support neurogenesis and synaptic plasticity. Exercise (both aerobic and resistance) appears to maintain or increase BDNF levels in HD patients and may therefore represent a potential mechanism explaining the beneficial effects of exercise on cognitive function [55,56]. Additionally, exercise reduces systemic inflammation and oxidative stress [56], both of which are strongly implicated in cognitive decline among dialysis patients, while also improving vascular health, glucose regulation, sleep quality, and increasing the removal of uremic toxins [18,19,57], factors known to affect cognitive function [19]. Taken together, these adaptations contribute to improved cognitive function in this high-risk population.

The findings of this review are consistent with prior reviews and emerging clinical trials of the effect of exercise on cognitive function in HD patients. In particular, it is generally supported that exercise interventions improve cognitive function in HD patients, regardless of contextual setting, with both interdialytic and intradialytic designs being associated with statistically significant improvements in cognitive function [53,54], especially in adults under 65 years of age, and when performed for at least 30 min, thrice weekly over 16 weeks [54]. However, it is generally the case that participant adherence is greater during intradialytic interventions, largely due to factors such as patient convenience and exploiting unavoidable captive treatment time during an established routine [53,54]. This may partly explain the statistically significant improvement in cognitive function scores reported by high adherence studies included in this review, within a considerably shorter intervention timeframe than previously reported. This review further extends previous findings through the highlighting of variation, both with respect to cognitive assessment tools and exercise intervention characteristics, especially with regard to frequency, intensity, duration, and type.

### 4.1. Limitations

Despite the strengths of this review, the results should be interpreted in light of certain limitations. Firstly, only a relatively small number of eligible studies were included in this review, two of which had a small sample size, which may have limited the statistical power and the generalizability of outcomes. Methodological concerns include the inability to conceal treatment allocation and adequately blind study participants, providers, and assessors to treatment group assignments across the majority of included studies, which can introduce bias. There was also a pronounced heterogeneity amongst the reported cognitive outcome measures and exercise intervention protocols, which complicates direct comparison across studies. Moreover, in the current systematic review, we used the Jadad scale to assess the risk of bias. However, future updates of the review could incorporate RoB 2 to provide greater methodological detail. Furthermore, this review was not registered in an international systematic review registry. Finally, most included studies were of short-term interventions; the absence of data from longer-term outcomes further limits the strength and generalizability of the evidence.

### 4.2. Implications for Future Research

Future research should employ larger sample sizes to improve statistical power and design interventions of longer duration (i.e., ≥6 months) with extended follow-ups to assess the sustainability of associated cognitive benefits. Studies must adopt a rigorous methodology, especially adhering to robust randomization, blinding, and allocation concealment practices to minimize bias. Furthermore, it is necessary to standardize the reporting of intervention characteristics—especially exercise frequency, intensity, duration, and type (aerobic, resistance, or hybrid)—alongside global and domain-specific cognitive outcome measures, to reduce between-study heterogeneity and facilitate meta-analytical syntheses, informing clinical guidelines.

## 5. Conclusions

The results of this review indicated a moderate beneficial effect on global and domain-specific cognitive scores, such as executive function and alertness. Clinically significant improvement is most likely after at least 12 weeks of moderate to high intensity intradialytic aerobic exercise, performed for 30–45 min, three times per week. However, future research employing larger sample sizes, over longer periods (≥6 months), and standardized reporting of exercise characteristics and cognitive outcome measures is required to inform guidelines.

## Figures and Tables

**Table 1:** PICOS item generated keywords.

PICOS Item	Keyword
Patient population	Hemodialysis patients
Interventions (exposures)	Exercise
Comparator (control) group	Standard care
Outcomes	Cognitive function
Study design	Randomized controlled trial

**Table 2:** Search algorithm input for each specific electronic database.

Database	Final Search Algorithm
PubMed	(((((hemodialysis [Title/Abstract]) OR (haemodialysis [Title/Abstract])) OR (dialysis [Title/Abstract])) OR (dialy* [Title/Abstract])) AND (((((“physical activity” [Title/Abstract]) OR (“physical exercise” [Title/Abstract])) OR (exercise* [Title/Abstract])) OR (activit* [Title/Abstract])) OR (fitness [Title/Abstract]))) AND (((cognition [Title/Abstract]) OR (cognitive [Title/Abstract])) OR (cognit* [Title/Abstract]))
EBSCO	AB (hemodialysis or haemodialysis or dialysis or dialy*) AND AB (physical activity or exercise or fitness or physical exercise or activit*) AND AB (cognition or cognitive function or cognitive performance or cognitive abilities or cognitive ability or memory or cognit*)
SCOPUS	((TITLE-ABS-KEY (hemodialysis) OR TITLE-ABS-KEY (haemodialysis) OR TITLE-ABS-KEY (dialysis) OR TITLE-ABS-KEY (dialy*))) AND ((TITLE-ABS-KEY (“physical activity”) OR TITLE-ABS-KEY (“physical exercise”) OR TITLE-ABS-KEY (exercise*) OR TITLE-ABS-KEY (activit*) OR TITLE-ABS-KEY (fitness)))AND ((TITLE-ABS-KEY (cognition)OR TITLE-ABS-KEY (cognitive) OR TITLE-ABS-KEY (cognit*)))

**Table 3:** Jadad score of likelihood of bias in included studies.

Study	Randomization	Blinding	Withdrawals/Dropouts	Score (0–5)
Ghildayal et al., 2025 [35]	++	0	+	3
Feng et al., 2025 [36]	++	0	+	3
Saputrana et al., 2024 [37]	0	0	+	1
Bogataj trial: Bogataj et al., 2023 [38] (A); Kren & Bogataj, 2023 [39] (B); Bogataj et al., 2024 [40] (C) *	++ (A, B, C)	0 (A, B, C)	+ (A, B, C)	3 (A, B, C)
Nakamura-Taira et al., 2021 [41]	+	0	+	2
McAdams-DeMarco et al., 2018 [42]	+	0	+	2
Stringuetta Belik et al., 2018 [43]	++	0	+	3

* Bogataj et al., 2023 [38] (A), Kren & Bogataj, 2023 [39] (B), Bogataj et al., 2024 [40] (C) are publications from the same trial cohort; scores are identical across reports. Scoring Key: each (+) is equal to one point; a (0) is equal to no points.

**Table 4:** NHLBI Quality Assessment of Controlled Studies.

Study ID	1	2	3	4	5	6	7	8	9	10	11	12	13	14	QR
Ghildayal et al., 2025 [35]	+	+	-	-	+	-	+	NR	+	NR	+	+	+	+	F
Feng et al., 2025 [36]	+	+	+	-	+	+	+	+	+	CD	+	+	-	CD	G
Saputrana et al., 2024 [37]	+	-	-	NR	NR	+	+	+	+	NR	+	-	-	-	F
Bogataj et al., 2023 [38] (A); Kren & Bogataj, 2023 [39] (B); Bogataj et al., 2024 [40] (C) *	+ (A, B, C)	+ (A, B, C)	NR (A, B, C)	- (A, B, C)	+ (A, B, C)	+ (A, B, C)	+ (A, B, C)	+ (A, B, C)	+ (A, B, C)	NR (A, B, C)	+ (A, B, C)	+ (A, B, C)	+ (A) NR (B, C)	+ (A, B, C)	G (A, B, C)
Nakamura-Taira et al., 2021 [41]	+	NR	NR	-	NR	+	-	+	+	+	+	+	NR	+	F
Stringuetta-Belik et al., 2018 [42]	+	+	+	-	NR	+	+	+	NR	+	+	-	NR	+	F
McAdams-DeMarco et al., 2018 [43]	+	NR	NR	NR	NR	+	+	+	+	NR	+	-	+	+	F

Abbreviations: CD, Cannot Determine; NA, Not Applicable; NR, Not Reported; QR, Quality Risk; (+), Yes (information included); (-), No (information not included) Score: G—Good, F—Fair, P—Poor. * Bogataj et al., 2023 [38] (A), Kren & Bogataj, 2023 [39] (B), Bogataj et al., 2024 [40] (C) are publications from the same trial cohort; scores are identical across reports.

**Table 5:** Summary characteristics and results of included studies.

Study	Year	Country	Sample Size	Age (Mean ± SD)/(IQR)	Sex (%Male)	Design	Intervention vs. Control	Measures	Outcomes
Ghildayal et al., 2025 [35]	2025	USA	ET: (n = 29) CT: (n = 31) ET + CT: (n = 35) SC: (n = 26)	ET: 60.4 (38.3–66.5) CT: 64.2 (56.2–69.9) ET + CT: 60.4 (52.0–70.3) SC: 61.5 (52.0–67.5)	ET: 66 CT: 65 ET + CT: 60 SC: 62	RCT	ET, CT, ET + CT vs. SC	TMTB-A TMT-A TMT-B MoCA DSST RAVLT	Between-group differences at 3 months compared to SC, presented as difference and (95% CI) TMTB-A (s) (n = 95) SC: reference ET: −15.8 (−54.7, 23.1) CT: 6.4 (−33.6, 46.4) ET + CT: −5.8 (−43.6,31.9) TMT-A (s) (n = 95) SC: reference ET: 11.4 (−2.7, 25.5) CT: 0.8 (−13.5, 15.1) ET + CT: 3.9 (−9.6, 17.5) TMT-B (s) (n = 95): SC: reference ET: −1.9 (−43.9, 40.2) CT: 11.4 (−31.7, 54.6) ET + CT: 4.1 (−36.4, 44.7) **MoCA (n = 77)** SC: reference ET: 0.7 (−1.1, 2.6) CT: 0.2 (−1.7, 2.0) **ET + CT: 2.1 (0.4, 3.9)** DSST (n = 77) SC: reference ET:7.4 (−4.8, 19.6) CT:3.8 (−8.5, 16.1) ET + CT: 2.9 (−8.8, 14.5) RAVLT (n = 79) SC: reference ET: −0.3 (−1.1, 0.6) CT: 0.2 (−0.6, 1.1) CT + ET: 0.3 (−0.5, 1.1)
Feng et al., 2025 [36]	2025	China	ET: (n = 28) SC: (n = 30)	ET: 57.50 ± 11.6 SC: 56.10 ± 12.07	ET: 61 SC: 67	RCT	ET vs. SC	MoCA	Within-group change (mean ± SD) **ET: from 24.21 ± 3.17 to 28.36 ± 1.75; t = −9.257, *p* < 0.001** SC: from 23.37 ± 4.19 to 24.37 ± 3.94; t = −1.888, *p* = 0.069 Interaction group x time: **t = 5.047, *p* < 0.001**
Saputrana et al., 2024 [37]	2024	Indonesia	ET: (n = 9) SC: (n = 9)	ET: 36.56 ± 4.61 SC: 39.44 ± 5.36	ET: 56 SC: 44	RCT	ET vs. SC	MoCA	Within-group change **ET: 22.56 ± 2.65 to 27.56 ± 2.29; ES = 1.88, *p* < 0.001** SC: 23.44 ± 2.29 to 24.00 ± 2.64; *p* = 0.468 Between-group differences **ET: 4 ± 2.64** SC: 1 ± 2.18 ***p* = 0.001**
Bogataj et al., 2023 [38] (A); Kren & Bogataj, 2023 [39] (B); Bogataj et al., 2024 [40] (C) *	2023/24	Slovenia	EXP (n = 22) CON (n = 21)	EXP: 65.7 ± 9.7 CON: 67.2 ± 12.5	EXP: 54 CON: 77	Single-Blind RCT	ET + CT vs. SC	(A) TAP Test	Within-group changes in (mean and 95% CI) Alertness: EXP: 43.3 (−8.7 to 95.3), *p* = 0.098, ES: +0.37 **CON: −26.7 (−52 to −1.4), *p* = 0.040, ES: −0.48** Selective attention: EXP: 20.4 (−22.1 to 62.9), *p* = 0.329, ES: +0.21 CON: −32.1 (−90 to 26.6), *p* = 0.267, ES: −0.25 Divided attention: EXP: 15.2 (−18.1 to 48.5), *p* = 0.352, ES: +0.21 CON: 16.1 (−36.6 to 68.7), *p* = 0.530, ES: +0.12 Between-group changes in Alertness: **(F(1,41) = 6.15, *p* = 0.017, η^2^ = 0.13)** Selective attention: (F(1,41) = 2.31, *p* = 0.136, η^2^ = 0.53) Divided attention: (F(1,41) = 0.001, *p* = 0.977, η^2^ = 0.00)
								(B) SDMT MoCA	Time × group interaction effect in favor of EXP group **SDMT: *p* < 0.001; η^2^ = 0.267** **MoCA: *p* < 0.001; η^2^ = 0.266I**
								(C) TMT-A TMT-B TMTB_A	Within-group changes: TMT-A (s): **EXP: −3.6 ± 5.5 (−6.1 to −1.2), *p* = 0.006, ES: 0.12** **CON: 6.6 ± 10.5 (1.8 to 11.3), *p* = 0.009, ES: 0.17** TMT-B (s): **EXP: −14 ± 17.2 (−21.7 to −6.4), *p* < 0.001, ES: 0.19** **CON: 7.0 ± 13.4 (0.9 to 13.1), *p* = 0.026. ES: 0.08** TMTB-TMTA (s): **EXP: −10.4 ± 15.2 (−17.1 to −3.7), *p* = 0.004, ES: 0.20** CON: 0.4 ± 17.9 (−7.7 to 8.6), *p* = 0.914, ES: 0.01 Time × group interaction effect for EXP group: **TMT**-**A: (F** [1,43] **= 16.218, *p* < 0.001, η^2^ = 0.283)** **TMT**-**B: (F** [1,43] **= 19.944, *p* < 0.001, η^2^ = 0.327)** **TMT_B—TMT_A: (F [1,43] = 4.606, *p* = 0.038, η^2^ = 0.101)**
Nakamura-Taira et al., 2021 [41]	2021	Japan	ET (n = 21) SC (n = 21)	ET: 74.9 ± 2.23 SC: 72.57 ± 2.26	ET: 33 SC: 71	Quasi-Cluster RCT	ET vs. SC	MoCA	Within-group change (AMS ± SE) EXP: 18.45 ± 0.63 to 18.87 ± 0.71 CON: 18.48 ± 0.77 to 18.09 ± 0.94 Comparison between groups ES: −0.13 (95% CI: −0.74 to 0.48) *p* > 0.05
Stringuetta Belik et al., 2018 [43]	2018	Brazil	ET (n = 15) SC (n = 15)	ET: 50.3 ± 17.24 SC: 57.8 ± 15.01	ET: 47 SC: 53	RCT	ET vs. SC	MMSE	Within group × time change **EXP: 24.0 ± 3.00 (baseline) to 26.4 ± 2.92 (post intervention); *p* < 0.001** CON: 22.4 ± 4.98 (baseline) to 23.0 ± 5.09 (post intervention); *p* > 0.050 Interaction between group × time ***p* = 0.001**
McAdams-DeMarco et al., 2018 [42]	2018	USA	ET: (n = 6) CT: (n = 7) SC: (n = 7)	ET: 48.0 ± 7.0 CT: 48.9 ± 12.2 SC: 55.0 ± 9.7	ET: 67 CT: 29 SC: 100	RCT	ET, CT vs. SC	3MS TMT-A TMT-B TMTB-A	Within-group change (mean ± SD) 3MS SC: −0.1 (7.1); *p* = 0.96 ET: 4.3 (5.4); *p* = 0.17 TMTA SC: 15.0 (25.8); *p* = 0.055 ET: −2.5 (9.3); *p* = 0.77 TMTB **SC: 47.4 (45.7); *p* = 0.006** ET: −8.9 (24.4); *p* = 0.63 TMTB—TMTA SC: 31.7 (47.8); *p* = 0.052 ET: −3.2 (31.2); *p* = 0.86 Between-group change (mean and 95% CI) (SC vs. ET) 3MS: 4.48 (95% CI: −4.27 to 13.22; *p* = 0.30 TMTA: −17.48 (95% CI: −41.18 to 6.22; *p* = 0.14) **TMTB: −56.21 (95% CI: −105.86 to −6.56; *p* = 0.03)** TMTB-TMTA: −34.93 (95% CI: −85.43 to 15.56; *p* = 0.16)

Abbreviations: EXP, experimental group; CON, control group; SC, standard care (control) group; ET, exercise training group; CT, cognitive training group; ET + CT, combined exercise and cognitive training intervention group; TAP Test, Test of Attentional Performance; MoCA, Montreal Cognitive Assessment; SDMT, Symbol Digit Modality Test; 3MS. Modified Mini Mental State Exam; TMTA, Trail Making Test A; TMTB, Trail Making Test B; TMTB-A, TMTB score minus TMTA score; MMSE, Mini Mental State Exam; DSST, Digit Symbol Substitution Test; RAVLT, Rey Auditory Verbal Learning Test; CI; Confidence Intervals; SD, standard deviation; AMS, adjusted mean score; SE, standard error; ES, effect size; CD, cannot determine; F, frequency (of intradialytic exercise training sessions); I, intensity (of intradialytic exercise sessions); T, time (intervention duration in weeks); T, type (of intradialytic exercise/mode of delivery); min., minutes; wks., weeks. In “**BOLD**” are statistically significant outcome measures. * Bogataj et al., 2023 [38] (A), Kren & Bogataj, 2023 [39] (B), and Bogataj et al., 2024 [40] (C) are reports from the same randomized controlled trial study.

**Table 6:** Summary of intradialytic exercise intervention characteristics of included studies.

Study	F.I.T.T. Principles: Frequency (Sessions/Week), Intensity (Rating), Time (Training Session Duration and Intervention Length), and Type (Exercise Modality)			
	Frequency	Intensity	Time	Type
Ghildayal et al., 2025 [35]	3	Moderate–High	30 min/12 wks.	Aerobic
Feng et al., 2025 [36]	3	Low–Moderate	30–40 min/12 wks.	Resistance
Saputrana et al., 2024 [37]	2	Low–Moderate	30 min/12 wks.	Aerobic
Bogataj et al., 2023/24 [38,40]	3	Moderate–High	30 min/12 wks.	Aerobic
Nakamura-Taira et al., 2021 [41]	3	Low	25–30 min/24 wks.	Resistance
Stringuetta Belik et al., 2018 [43]	3	Moderate–High	30–45 min/16 wks.	Aerobic
McAdams De-Marco et al., 2018 [42]	3	CD	20 min/12 wks.	Aerobic

Abbreviations: CD, cannot determine; min, minutes; wks., weeks.

## Data Availability

No new data were created or analyzed in this study. Data sharing is not applicable to this article.
